# Use of *Drosophila* as an Evaluation Method Reveals *imp* as a Candidate Gene for Type 2 Diabetes in Rat Locus *Niddm22*

**DOI:** 10.1155/2015/758564

**Published:** 2015-03-02

**Authors:** Kurenai Kawasaki, Sawaka Yamada, Koki Ogata, Yumiko Saito, Aiko Takahama, Takahisa Yamada, Kozo Matsumoto, Hiroyuki Kose

**Affiliations:** ^1^Division of Natural Sciences, Department of Life Science, International Christian University, Mitaka, Tokyo 181-8585, Japan; ^2^Laboratory of Animal Genetics, Graduate School of Science and Technology, Niigata University, Niigata 950-2181, Japan; ^3^Department of Animal Medical Sciences, Faculty of Life Sciences, Kyoto Sangyo University, Kyoto 603-8555, Japan

## Abstract

Type 2 diabetes (T2D) is one of the most common human diseases. QTL analysis of the diabetic Otsuka Long-Evans Tokushima Fatty (OLETF) rats has identified numerous hyperglycemic loci. However, molecular characterization and/or gene identification largely remains to be elucidated due mostly to the weak genetic variances contributed by each locus. Here we utilized *Drosophila melanogaster* as a secondary model organism for functional evaluation of the candidate gene. We demonstrate that the tissue specific knockdown of a homologue of *igf2bp2* RNA binding protein leads to increased sugar levels similar to that found in the OLETF rat. In the mutant, the expression of two of the insulin-like peptides encoded in the fly genome, *dilp2* and *dilp3*, were found to be downregulated. Consistent with previous reports of *dilp* mutants, the *imp* mutant flies exhibited an extension of life span; in contrast, starvation tolerance was reduced. These results further reinforce the possibility that *imp* is involved in sugar metabolism by modulating insulin expression.

## 1. Introduction

The world health organization (WHO) currently estimates that over 300 million individuals worldwide suffer from diabetes, 90% being type 2 diabetes (T2D) [1]. T2D, the primary feature of which is a state of chronic elevation of plasma glucose levels, is a polygenic disease that is caused by a metabolic and hormonal imbalance between insulin secretion from pancreatic *β*-cells and insulin resistance in peripheral tissues. Much effort has been devoted to the development and characterization of monogenic diabetes animal models, which have led to significant advancements in our understanding of the genetic basis of glucose/lipid metabolisms as well as the molecular pathogenesis of complications [2]. In spite of the progress, the importance of polygenic or spontaneous diabetes models is not diminished because the majority of genetic variations that are causative for a complex disease are not amorphic, but hypomorphic [3–5]. However, the importance of spontaneous diabetes models has been relatively underestimated owing to the difficulty of positional cloning [6].

The OLETF rat is one of the most studied strains by virtue of its similarity to a particular human population marked by propensity for disorders of glucose metabolism [7]. Traditional genetic analysis in the OLETF has been based on mapping QTL using microsatellite markers, followed by genetic isolation of QTL in congenic strains [8]. Recently there have been several studies reporting the successful positional cloning of QTLs by further extensive fine mapping of congenic strains [3]. However, in these cases the LOD scores of the QTL are relatively high (above 6.0) and consistently the identified mutations led to a more than twofold increase or reduction in expression levels [3, 4]. Thus, the search for genetic factors for polygenic traits remains to be a formidable challenge, especially for those whose LOD scores are not very high. Ideally rodent models should be used for functional probing of the candidate genes, yet the screening of a large number of genes is considered to be intractable with current techniques.

*Drosophila melanogaster* has progressively been recognized as the most feasible nonmammalian model for metabolic diseases [9, 10]. The* Drosophila* genome encodes eight insulin-like peptides and the backbone of the insulin/IGF-like signaling (IIS) pathway is highly conserved in comparison to that of vertebrates. Furthermore, physiological roles of the IIS pathway, including growth, lifespan, stress resistance, and metabolism, are also analogous across animal kingdom, making* Drosophila* a potential alternative agent for functional evaluation of the genes whose candidacy is suggested in other systems. The use of* Drosophila* as an evaluation method will be useful at least for those genes whose orthologs are encoded by the fly genome. One of the hyperglycemic QTLs identified in our previous studies is intriguing in terms of its association with obesity (see Section 4) and it is worth further investigation.

In the current study, prior to full-scale screening, we chose to focus on another QTL,* Niddm22* (*Nidd4/of* as our original nomenclature), because of the presence of a strong candidate gene.* Niddm22* is a region of 35.4 cM, corresponding to a physical distance of 24 Mbp, on the rat chromosome 11 [8]. Several human linkage studies reported metabolic QTLs in its syntenic region [11, 12]. According to the Ensemble database (release 73), 161 genes are annotated in this rat chromosomal segment [13]. Among those, 80 genes have fly orthologues. Here we focused on* imp*, a homolog of vertebrate* igf2bp2*, because the association studies identified an SNP within the locus to be linked not only to the diabetic phenotype but also to other diabetes-related traits such as fasting glucose, glucose AUC (area under curve), and Cederholm index [14–16].

## 2. Materials and Methods

### 2.1. Fly Stocks

All fly stocks were reared at 25°C on a standard yeast (4%, w/v), corn meal (8%, w/v), glucose (10%, w/v), and agar medium, under 12 h:12 h light:dark conditions unless otherwise stated. The following fly stocks were used: UAS-*imp-*RNAi (v20321) from the Vienna Drosophila RNAi Center, Imp protein trap strain (number 110921) from the Drosophila Genetic Resource Center at Kyoto Institute of Technology. Additional fly stocks were generously provided by the Drosophila community: elav-GAL4 and UAS-Dcr2 from Yasushi Hiromi [17–19], dilp2-Gal4 from Takashi Nishimura [20, 21]. Using standard fly genetics, UAS-Dcr2 and UAS-*imp*-RNAi were intercrossed into one strain in order to enhance the effect of RNA interference. Here UAS-*imp*-RNAi; UAS-Dcr-2 is referred to as UAS-*imp* ^RNAi^.

### 2.2. Metabolic Studies

Whole-fly or hemolymph trehalose was measured by a Trehalose Assay Kit (Megazyme, K-TREH). For whole-fly preparation, 10 larvae were collected and briefly rinsed in Ringer's solution. The larvae were homogenized by vigorous shaking in the presence of Zirconia beads (NIKKATO, *φ* 0.8 YTZ Ball). The resultant homogenate was heated at 70°C for 5 min and centrifuged at 12000 rpm for 5 min and the resultant supernatant was used for subsequent measurements. Hemolymph was prepared as previously described [22]. Briefly, 10 third instar larvae were pricked with a tungsten needle and transferred to a microfuge tube which had been pierced in the bottom, which was then piggybacked and centrifuged for 5 min at 4°C, 7,000 rpm. The resultant supernatant or hemolymph was used for subsequent measurements. Protein quantity was determined by Quant-iT Protein Assay Kits (Invitrogen).

### 2.3. Lifespan Assay

Lifespan studies were performed as previously mentioned with modifications [23]. For both fed and starved samples, three to ten virgin males and virgin females with approximately 1 : 1 ratio were placed in a single plastic vial. For starvation, the vial contained a piece of filter paper moisturized with distilled water. Flies were transferred to fresh medium or moisture vials every four to five days, and deaths were scored three times per week. The number of live individuals was recorded until all flies died.

### 2.4. q-PCR

Total RNA was extracted from 25 whole larvae in TRIzol reagent (Invitrogen). One microgram of total RNA was used for reverse transcription with iScript Select cDNA Synthesis Kit (Bio-Rad) by using oligo(dT) primer. q-PCR was performed on a MiniOpticon real-time PCR System (Bio-Rad) using iQ SYBR Green Supermix (Bio-Rad). Primers used for Q-RT-PCR are summarized in Table S1 available online at http://dx.doi.org/10.1155/2015/758564 [24, 25].

### 2.5. Statistical Analyses

For all experiments, error bars represent SEM, and *P* values are the results of ANOVA followed by post hoc analyses using Scheffe's test.

### 2.6. Microscopy

Fluorescent and bright field images were taken using an Axio 200 microscope (Zeiss).

## 3. Results

### 3.1. CNS Specific* imp* Knockdown Resulted in Hypertrehalosemia

**Figure 1:**
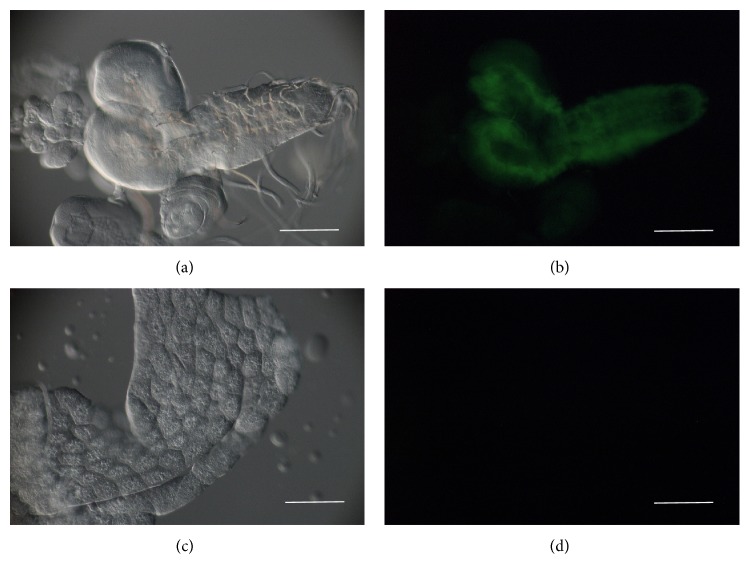
Expression pattern of the GFP-tagged protein trap.* imp* expression in third instar larva was examined using a protein trap strain ZCL0310, which expresses GFP-Imp chimeric protein in endogenous* imp*-expressing tissues. Bright field (a, c) and intrinsic GFP (b, d) images are shown. The GFP signal was detected in the central nervous system (CNS) (a, b), but only in a subset of neurons within the CNS. In contrast, GFP-Imp was not confirmed in the fat body (c, d). Bar, 100 *μ*m.

**Figure 2:**
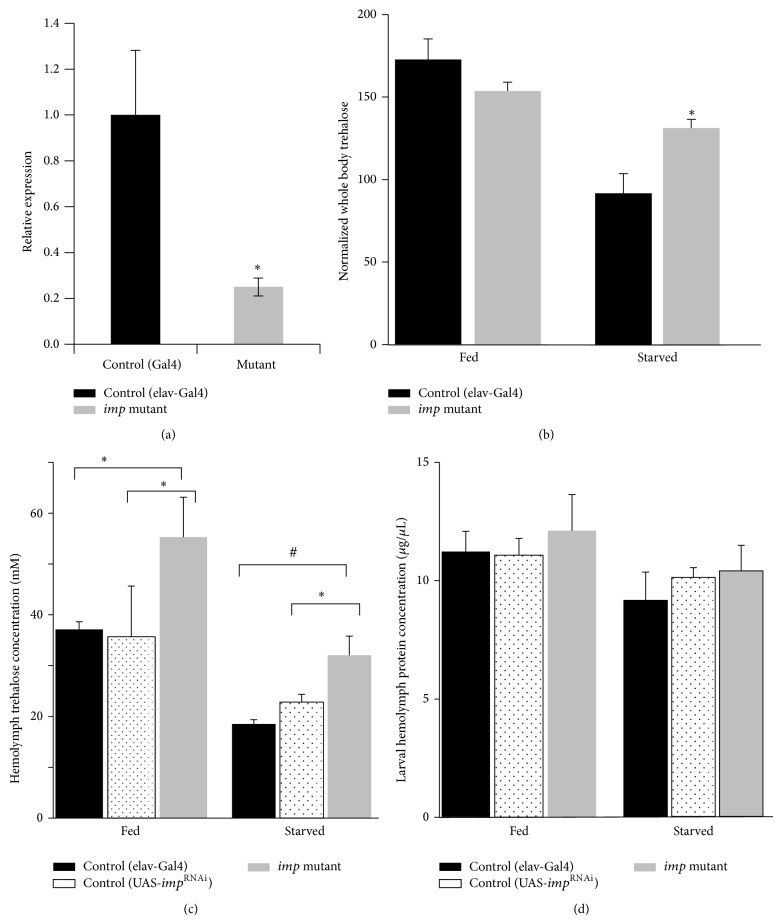
Abnormal sugar metabolism is observed in the* imp* knockdown larva. (a)* imp* transcript was analyzed by q-PCR in larvae of the indicated genotypes. It was confirmed that the CNS specific knockdown of* imp* led to reduction of* imp* to about one-fifth of that of the control strain. (b) Normalized trehalose in whole larvae homogenized preparation was compared in order to examine the overall carbohydrate metabolism with protein concentration as an internal reference. The* imp* mutant shows a delay in trehalose usage in the fasting condition (7-hour fast, *n* = 5). (c) Hemolymph trehalose concentration is increased in the* imp* mutant for both fed and starved condition. The difference is more prominent after 15-hour fasting (*n* = 6). ^*^ *P* < 0.05. ^#^ *P* < 0.01 (d) In contrast, hemolymph protein concentration was unchanged for both fed and starved condition.

Previous studies showed that* imp* is expressed in the central nervous system and pole cells during embryonic development and germ cells in adults [26–28]. In order to examine the expression pattern of* imp* in larvae, we analyzed a protein trap strain, ZCL0310 [28]. In the third instar wandering larvae, the expression was exclusively detected in the central nervous system (CNS) (Figure 1). In contrast,* imp* is not expressed in other metabolically crucial tissues, including body wall muscle, fat body, gut, and oenocytes (Figure 1). Next we produced an CNS specific* imp* knockdown strain. The mutant had normal hatching rate and developmental growth. No morphological defect was observed. We confirmed that, in the third instar larvae,* imp* expression was reduced to about 20% of that of control (Figure 2(a)). Hemolymph was extracted from the third instar larvae that were immersed in the food medium (fed state). We also tested hemolymph from larvae starved for 15 hours (starved state). In both cases, the trehalose levels were significantly higher for the* imp* mutant compared with either control strain (Figure 2(c)). In contrast, no difference was observed among these strains for protein levels in either fed or starved condition. In order to examine the effect of* imp* knockdown mutation on total trehalose levels, whole-fly trehalose that is normalized by total protein levels was compared. In the starved state, the total amount of trehalose was higher than the control (Figure 2(b)). Because in our QTL analysis* Niddm22* locus was identified as fasting hyperglycemic QTL, the observation of a more prominent effect on the starved state implies gene candidacy.

### 3.2. *dilp* Expression Is Downregulated in* imp* Knockdown Mutant

**Figure 3:**
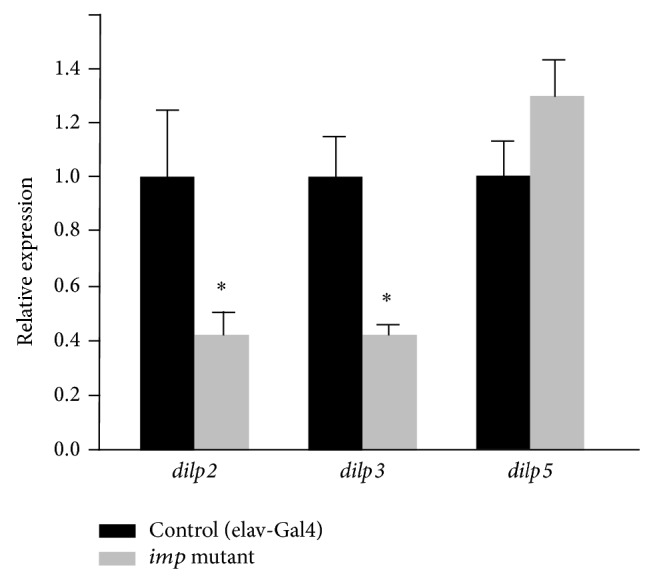
*dilp2* and* dilp3* expression are significantly reduced in the* imp* mutant larvae. q-PCR analysis was conducted to examine the* dilp* expression on the third instar larva in the fed state.* dilp2* and* dilp3*, but not* dilp5*, were reduced to less than the half of the control strain. ^*^ *P* < 0.05.

We examined the expression of a subset of* dilp* genes that are crucial for carbohydrate metabolism [29, 30]. The expression levels of* dilp2* and* dilp3*, but not* dilp5*, were significantly reduced in the* imp* mutant larva (Figure 3). Imp belongs to a family of mRNA-binding proteins that play an important role in RNA localization, stability, and translation. RNA binding is mediated by highly conserved KH domains [31]. One of the most characterized KH domains, KH3 of Nova, recognizes a single UCAY element in the context of a 20-base hairpin RNA [32]. We found 2, 6, and 2 consensus motifs in* dilp2*,* dilp3*, and* dilp5* mRNA, respectively (Table S2), leading us to the hypothesis that Imp may posttranscriptionally control the translation of* dilps* by direct binding. To test this, we knocked down the function of* imp* only in insulin-producing cells (IPCs) in which the three* dilp* isoforms are most exclusively expressed. However, the levels of* dilps* (*dilp2*,* dilp3*, and* dilp5*) or* imp* were unchanged and no hypertrehalosemia was observed (Figure S1). Furthermore, immunostaining revealed no apparent* imp* expression in IPCs (Figure S2). All of these results suggest that* imp* influences subtypes of* dilp* expression in a cell-non-autonomous manner.

### 3.3. *imp* Knockdown Resulted in Longer Lifespan and Reduced Starvation Tolerance

**Figure 4:**
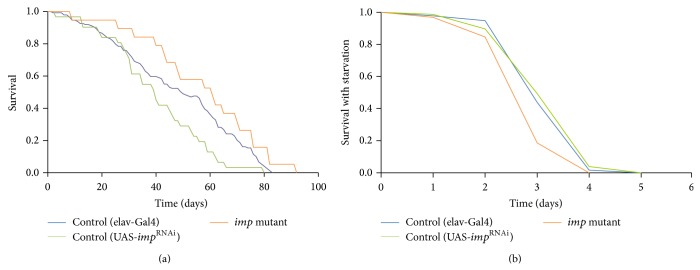
*imp* knockdown mutant exhibited longer lifespan and reduced starvation resistance. (a) Survival of elav-Gal4 virgin flies (blue, *n* = 149), UAS-*imp* ^RNAi^ (green, *n* = 31), and* imp* mutant [elav-Gal4; UAS-*imp* ^RNAi^] (orange, *n* = 19). Median lifespans are as follows: elav-Gal4, 51 days; UAS-*imp* ^RNAi^; UAS-Dicer2, 40 days; elav-Gal4; UAS-*imp* ^RNAi^ 62 days, *P* < 0.038 versus elav-Gal4, *P* < 0.0004 versus UAS-*imp* ^RNAi^ (log-rank test). (b) Survival of virgin flies during starvation: elav-Gal4 virgin flies (blue, *n* = 303), UAS-*imp* ^RNAi^ (green, *n* = 77), and* imp* mutant [elav-Gal4; UAS-*imp* ^RNAi^] (orange, *n* = 188). Average lifespans are as follows: elav-Gal4, 3.38 ± 0.04 days; UAS-*imp* ^RNAi^  3.42 ± 0.05 days;* imp* mutant [elav-Gal4; UAS-*imp* ^RNAi^] 3.00 ± 0.09 days, *P* < 0.0001 versus elav-Gal4, *P* < 0.0001 versus UAS-*imp* ^RNAi^ (log-rank test).

There are numerous reports that link the IIS pathway to lifespan or aging [24]. Our results so far suggest that the IIS signaling activity may be chronically lowered in the* imp* mutant. Consistently the* imp* knockdown mutant exhibited a significant increase in average and maximum lifespan over that of control flies (Figure 4(a)). Previous studies reported that the IPC-ablated flies were slightly starvation resistant [24, 33]. The extended lifespan is usually considered to be the result of enhanced stress resistance. However, the longevity on starvation of* imp* mutants is significantly lower than that of the control strains (Figure 4(b)), suggesting that* imp* may be involved in stress response regulation independent of* dilp* activity.

It is also widely known that IIS signaling pathway plays an essential role in the control of cell size and growth [30, 34]. The loss of* dilp2*, but not* dilp3* or* dilp5*, reduces body weight [30]. Body size and weight of the* imp* knockdown strain are unchanged (Figure S3). This may be because the remaining* dilp2* expression is sufficient to maintain normal growth, or other members of* dilps* that control growth compensate for the effect of* dilp2* downregulation.

### 3.4. Polymorphisms of* igf2bp2* Locus and Expression Analysis

Next we sequenced the coding region of* igf2bp2*, a rat orthologue of* imp* for the OLETF and F344 rat. There is one SNP in the fourth exon; however this SNP is a synonymous substitution (Table S3). Furthermore, in the tissue examined, our q-PCR analysis failed to detect any difference in the expression levels between the two strains. Further studies will be necessary for establishing the causality of the* igf2bp2* in the OLETF rat.

## 4. Discussion

In the present study we demonstrated that the examination of the homologous gene provides us with a unique opportunity to search for novel metabolic genes. We tested* Niddm22* here partly because our aim was to establish the methodology; we now wish to tackle other novel QTLs.

Previously we demonstrated that one of the hyperglycemic QTLs,* Niddm20* (*Nidd2/of* as our original nomenclature), located on chromosome 14 of the OLETF rat, is quite unique for the following reasons: (1) its LOD score (4.07 for 30 min postprandial plasma glucose) is one of the highest among the other QTLs [35, 36]; (2) there is a strong epistasis with other QTLs [37]; (3) most importantly it interacts with the obese condition: the congenic strain exhibits more severe diabetic symptoms when combined with either genetically or nutritionally induced obesity [38, 39]. From the clinical point of view, identification of causative genes in such QTL has to be given higher priority. We further fine-mapped the region to discover that* Niddm20* is composed of at least two narrower loci, each of which is localized at proximal and distal ends of the QTL region [40]. The analysis of subcongenic strains of* Niddm20* showed that the exclusion of either locus from the original* Niddm20* region resulted in the loss of the hyperglycemic phenotype, suggesting an epistatic relationship between the subloci. Within the syntenic region of the human genome, neither diabetic QTL has been reported nor have any of the T2D susceptibility genes been mapped [41]. Therefore, elucidation of the molecular nature of* Niddm20* may provide novel opportunity for understanding human T2D. According to the Ensemble database, there are 62 genes annotated within the proximal 10Mb of* Niddm20*, none of which has been implicated with T2D. Our aim is to utilize* Drosophila* for functional evaluation of those candidate genes. A similar attempt was recently reported elsewhere [42] and it is hoped that* Drosophila* as a secondary model will help to find novel diabetic genes.

*igf2bp2* has been implicated by genome-wide association studies as a candidate susceptibility gene for T2D [14, 15]. Several association studies correlated* igf2bp2*-SNPs more with reduced pancreatic *β*-cell activity than insulin resistance [43, 44]. However, the SNP is found in the second intron and the mechanism by which this susceptibility is engendered is unknown. Dai et al. reported that* igf2bp2* mRNA is promoted by phosphorylation of Igf2bp2 by mTOR [45]. Another study indicated that Igf2bp2 directly binds to laminin-*β*2 mRNA and regulates its translation in a glucose concentration-dependent manner in the podocyte [46]. Several* Drosophila* studies also investigated the role of* imp* in the context of mRNA translocalization as well as translational regulation [26, 27, 47]. In the tests* imp* plays a crucial role in the aging of germ line stem cells (GSC), implicating a possible connection with the extended lifespan observed in the* imp* knockdown flies [48].

Among the genes annotated in* Niddm22*, there are other candidate genes that are inferred to be involved in metabolic functions, including somatostatin [49], Ahsg [50], and Adipoq [51]. Adipoq is the only other gene that has been identified as a diabetes candidate gene by GWAS. Recently it was reported that an adiponectin receptor homologue is involved in carbohydrate metabolism in the fly; however its orthologous ligand is not encoded in the fly genome and the authentic ligand remains to be discovered [52].

In general* Drosophila* offers a convenient resource for providing a rapid, inexpensive* in vivo* test of gene function. In addition fly genetics could also be useful for understanding molecular mechanisms. Indeed some insulin pathway components have been identified or validated by* Drosophila* research [53]. Reduced* dilp* level in the* imp* mutant is consistent at least partially with mammalian studies. It is, however, important to notice that in this system the following: (1) genes can only be characterized for which there are functional homologues in fly and (2) findings of diabetes-like phenotypes may not be valid for vertebrates or humans. For example, even though many insulin signaling components are conserved between flies and mammals, there are as many as eight insulin-like genes in* Drosophila* and they are expressed in tissues of various developmental origins, such as* dilp2*,* dilp3*, and* dilp5* in neurons or* dilp6* in the fat body [54]. With that in mind, it is hoped that genetic screening using this strain as a platform might elucidate details of the molecular pathway.

## 5. Conclusions

In summary, we performed a functional analysis on one of the diabetic candidate genes derived from the OLETF rat. We showed that downregulation of* imp* led to a hypertrehalosemic condition in* Drosophila*. Although further studies will be necessary to confirm the causative relationship between* imp* and diabetes in the OLETF rat, our results indicate that* Drosophila* is a useful secondary model for examination of the mammalian diabetes model.

## Supplementary Material

List of primers that were used for q-PCR analysis. Primers for *gapdh1* and *imp* were designed by Primer3 .
