# Supplementary material for: Use of Drosophila as an Evaluation Method Reveals imp as a Candidate Gene for Type 2 Diabetes in Rat Locus Niddm22
Source: J Diabetes Res. 2015 Mar 2;2015:758564. doi: 10.1155/2015/758564 (PMC4363715; doi:10.1155/2015/758564)
Supplement: Supplementary file 1 — List of primers that were used for q-PCR analysis. Primers for gapdh1 and imp were designed by Primer3 . [file 758564.f1.pdf]

Table S1 - PCR Oligonucleotide Primers:

Name	Gene	Sequence	Reference
dGAPDH1.751-	*Gapdh1*	ATCGTCGAGGGTCTGATGAC	This Study
dGAPDH1.-994	*Gapdh1*	CGGACGGTAAGATCCACAAC	This Study
IMP-RA.E4/6.99-	*Imp*	GCGAAGAAGGCTTCATGTGT	This Study
IMP-RA.E4/6.-299	*Imp*	TGTGCACTGGTGTCTCTTCA	This Study
dlip2.Clements.F	*Dilp2*	GCGAGGAGTATAATCCCGTGAT	[54]
dlip2.Clements.R	*Dilp2*	GGATTGAGGGCGTCCAGAT	[54]
dlip3.Broughton.F	*Dilp3*	AGAGAACTTTGGACCCCGTGAA	[55]
dlip3.Broughton.R	*Dilp3*	TGAACCGAACTATCACTCAACAGTCT	[55]
dlip5.Broughton.F	*Dilp5*	GAGGCACCTTGGGCCTATTC	[55]
dlip5.Broughton.R	*Dillp5*	CATGTGGTGAGATTCGGAGCTA	[55]

Table S2 – K-homology (KH) domain consensus RNA binding motif:

>FBtr0076329 type=mRNA dilp2 670bp

TCGACCCGGCTCGACCCAACTTAATCCATTTGATCGTAAAGCAACCTAAGCA
GTAAACCCATAACCATGAGCAAGCCTTTGTCCTTCATCTCGATGGTGGCCGT
GATTTTGCTGGCCAGCTCCACAGTGAAGTTGGCCCAAGGAACGCTCTGCAG
TGAAAAGCTCAACGAGGTGCTGAGTATGGTGTGCGAGGAGTATAATCCCGT
GATTCCACACAAGCGCGCCATGCCCGGTGCCGACAGCGATCTGGACGCCCT
CAATCCCCTGCAGTTTGTCCAGGAGTTCGAGGAGGAGGATAACTCGATATC
GGAACCGCTGCGAAGTGCCCTCTTTCCTGGGAGCTATCTTGGGGGTGTACTC
AATTCCCTGGCTGAAGTCCGGAGGCGAACTCGCCAACGGCAAGGAATCGTG
GAGAGGTGCTGCAAAAAGTCCTGTGATATGAAGGCTCTGCGGGAGTACTGC
TCCGTGGTCAGAAATTAGGCCTCCTAATGCGAAAATCATTGACCCCAACTG
ACCTGGTCGACGCGATTATCTCTGGATCTGGTTCCAAACCAACCATGTGCAT
ATATACTACAATCGATGTTTTTACAGCTTGTTGCATGTTACTCTTTACGAAT
GATCGAAATGGATTAAATATATATTCTGCTTTAAGCTTTGGCAAACAATCGC

>FBtr0076373 type=mRNA dilp3 751bp

ACTTGGCAGACACATACTACACACTCCCCGGGGGATTCACGCATCCATACTT
AAACACCACTTCATCACTCATGGGCATCGAGATGAGGTGTCAGGACAGGAG
GATCCTGCTACCTAGCCTACTCCTACTAATCCTTATGATCGGCGGTGTCCAG
GCCACCATGAAGTTGTGCGGCCGCAAACTGCCCGAAACTCTCTCCAAGCTCT
GTGTGTATGGCTTCAACGCAATGACCAAGAGAACTTTGGACCCCGTGAACT
TCAATCAGATCGATGGCTTCGAAGACCGTTCCCTGCTGGAAAGACTGTTGA
GTGATAGTTCGGTTCAGATGCTCAAGACTCGACGTCTTCGGGATGGAGTCTT
CGACGAGTGTTGCCTGAAGTCGTGCACCATGGATGAGGTGCTGAGATATTG
TGCTGCCAAGCCGAGAACGTAAACCTCGTAAACCTATTAACCCAATGACGA
CAACTGCGATGATTGAAATGGAATGAAAGGACCCGATTGGGGAAAGCACTC
ACGTAATCATAGTTGTTAAGTCGTTATCGAAGCCTACTCAATTCCAACTTTG
GATTTATGATATATATGCACATGTAAGAGGGATGTATGCGCATAATTTATGA
TCTGAAATCAGAGACAGGCACGCGAAATGAATCGGAACACGGGATGTTATG
CATGGTAGATATGTATGATTGTGCGGGGCCAGAATACATCGCCTGGGTATA
AATTATTAAATAAATTATGTATTCAAACTGC

>FBtr0076371 type=mRNA dilp5 478bp

TCCAGCAGCAGTTCCAGCAAGGCAATGATGTTCCGCTCCGTGATCCCAGTTC
TCCTGTTCCTGATCCCGCTCCTGCTATCCGCCCAGGCCGCAAACTCGCTGCG
GGCTTGTGGCCCCGCCTTGATGGACATGCTGAGGGTTGCCTGTCCCAATGGA
TTCAATTCAATGTTCGCCAAACGAGGCACCTTGGGCCTATTCGATTATGAGG
ACCACTTGGCGGATTTGGATAGCTCCGAATCTCACCACATGAACTCACTGTC
GAGCATTCGGCGCGATTTTCGCGGCGTTGTCGACTCCTGTTGCCGCAAATCG
TGTTCCTTTTCCACGTTGAGGGCATACTGCGACTCCTAAAAATGCTGCGATA
CCTGGTCCATCAGTTTACATACATACATATATGAGACATGTTACGTAAAAGT
ATTAAGTATAAACTATATGCACGGCTTTGAAACTTGAAACTACAATAAATA
AGCGCAATTCG

In *dilp* mRNA sequences, there are 2, 6, 2 conserved binding motifs (TCAT[yellow], TCAC[blue]) in *dilp2*, *dilp3* and *dilp5*, respectively.

Table S3 – SNP in the coding region of *igf2bp2*

exon4 Ensemble.ape from 1 to 252

Alignment to

exon4 F344.ape-- Matches:252; Mismatches:0; Gaps:0; Unattempted:0

exon4 OLETF.ape-- Matches:251; Mismatches:1; Gaps:0; Unattempted:0

```
             *         *         *         *         *         *         *         *         *         *
  1>AGCTATTGAGAAGTTAAGTGGGCATCAGTTCGAGGACTACTCCTTCAAGATTTCCTACATCCCCGACGAAGAATTGAGCTCCCCTTCACCCCCTCATCGA>100
  1>AGCTATTGAGAAGTTAAGTGGGCATCAGTTCGAGGACTACTCCTTCAAGATTTCCTACATCCCCGACGAAGAATTGAGCTCCCCTTCACCCCCTCATCGA>100
  1>AGCTATTGAGAAGTTAAGTGGGCATCAGTTCGAGGACTACTCCTTCAAGATTTCCTACATCCCCGACGAAGAATTGAGCTCCCCTTCACCCCCTCATCGA>100

             *         *         *         *         *         *         *         *         *         *
101>GCCCGGGAACAAGGCCATGGCCCCGGGAGCTCTTCTCAGGCCAGACAGATTGACTTCCCGCTGAGGATCCTGGTCCCCACCCAGTTTGTTGGTGCCATCA>200
101>GCCCGGGAACAAGGCCATGGCCCCGGGAGCTCTTCTCAGGCCAGACAGATTGACTTCCCGCTGAGGATCCTGGTCCCCACCCAGTTTGTTGGTGCCATCA>200
101>GCCCGGGAACAAGGCCATGGCCCCGGGAGCTCTTCTCAGGCCAGACAGATTGACTTCCCGCTGAGGATCCTGGTCCCCACCCAGTTTGTTGGTGCCATCA>200

             *         *         *         *         *
201>TCGGAAAGGAGGGCTTGACCATAAAGAACATCACTAAACAGACCCAGTCCCG>252
201>TCGGAAAGGAGGGCTTGACCATAAAGAACATCACTAAACAGACCCAGTCCCG>252
201>TCGGAAAAGAGGGCTTGACCATAAAGAACATCACTAAACAGACCCAGTCCCG>252
```

In the exon 4 of *igf2bp2*, one SNP where G208 is replaced with A is found. This alteration does not result in amino acid change.

Figure S1

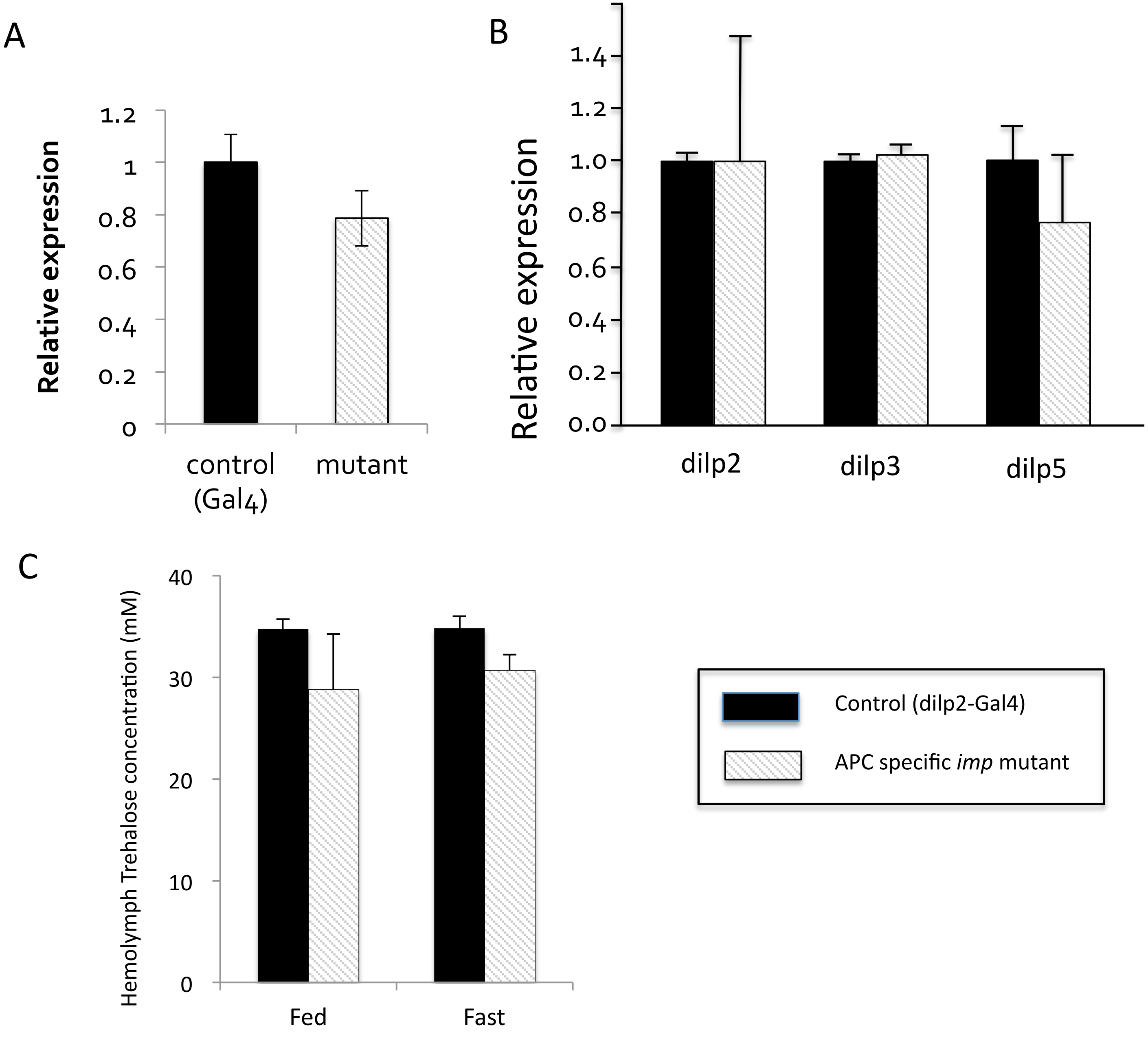

A
Relative expression
1.2
1
0.8
0.6
0.4
0.2
0
control (Gal4)
mutant
B
Relative expression
1.4
1.2
1.0
0.8
0.6
0.4
0.2
0.0
dilp2
dilp3
dilp5
C
Hemolymph Trehalose concentration (mM)
40
30
20
10
0
Fed
Fast
Control (dilp2-Gal4)
APC specific *imp* mutant

Figure S2

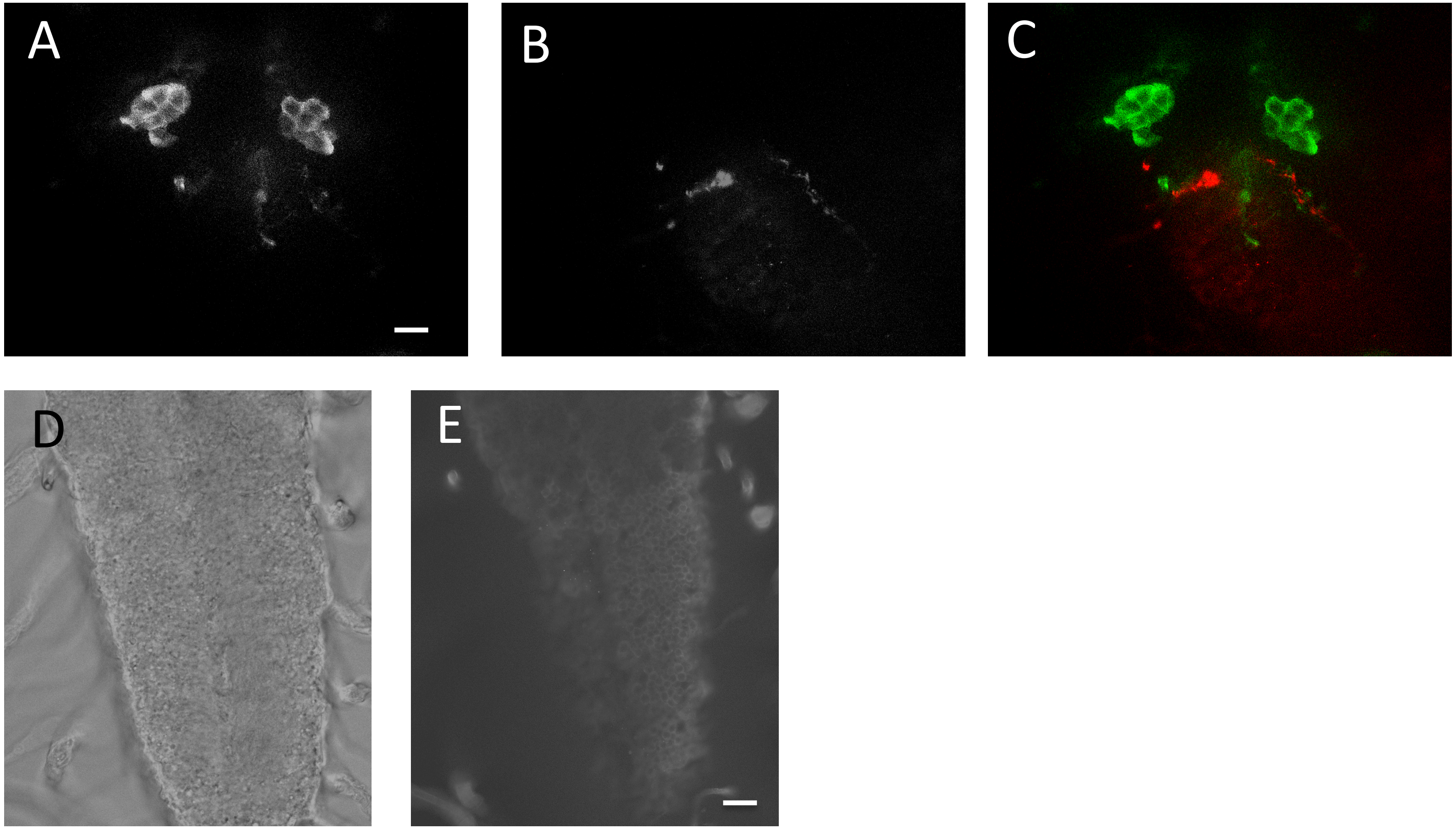

Figure S3

A

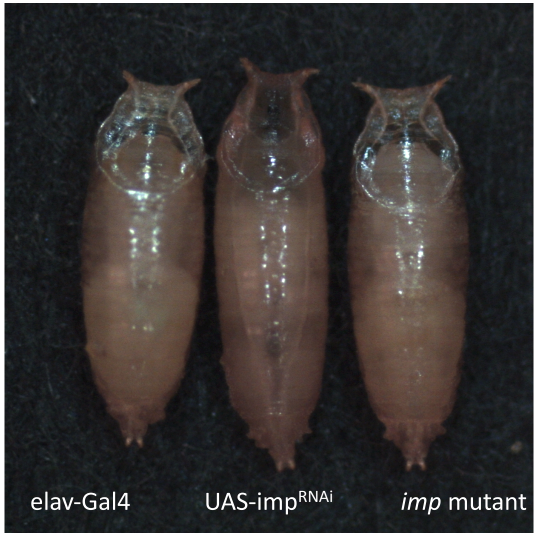
elav-Gal4
UAS-imp RNAi
imp mutant

B

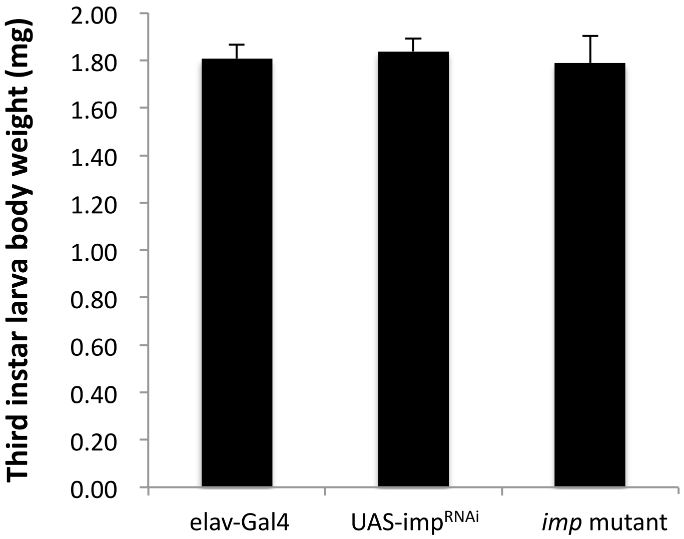
Third instar larva body weight (mg)
2.00
1.80
1.60
1.40
1.20
1.00
0.80
0.60
0.40
0.20
0.00
elav-Gal4
UAS-imp RNAi
imp mutant

Figure S1. It is unlikely that *imp* functions in a cell-autonomous manner**.** (A) IPC specific knockdown of *imp* was achieved by using dilp2-Gal4 as a driver strain. Unlike the pan-neuronal knockdown mutant, IPC specific knockdown of *imp* resulted in no difference in expression levels. (B) Similarly, transcription levels of none of the *dilps* showed differences in the IPC specific *imp* knockdown larvae. (C) Larval hemolymph trehalose levels were unchanged in either fed or fast condition (7 hour fasting, n=4).

Figure S2. Immunofluoresent analysis did not detect Imp protein in the soma of IPC. The larval CNS from the dilp2-Gal4;UAS-GFP was double-stained for GFP (A) and Imp (B). (C) is an overlay. Consistent with the Imp protein trap strain (Figure1B), Imp was expressed broadly in the nerve cord of the CNS. Rabbit polyclonal serum against Imp was generated against synthetic polypeptide (GSKLHAEQLDKNQR) (eurofins). Bars, 10 μm (A), 50μm (B).

Figure S3. Body size and body weight were unchanged for the *imp* mutant. No difference was observed for the size of pupae among elav-Gal4, UAS-imp^{RNAi}, and the *imp* mutant (A). Body weight of the third instar wandering larvae was also unchanged. p=0.87 vs. elav-Gal4, p=0.68 vs. UAS-imp^{RNAi} (n=10 for each group).
